# Putting It All Together: Postmortem Diagnosis of a Rare Ichthyosis Syndrome

**DOI:** 10.7759/cureus.38787

**Published:** 2023-05-09

**Authors:** Pragya Virendrakumar Jain, Jauntea Maxey, Michael W Lawlor, Lauren N Parsons

**Affiliations:** 1 Pathology, Medical College of Wisconsin, Milwaukee, USA; 2 Medicine, Medical College of Wisconsin, Milwaukee, USA

**Keywords:** phgdh mutation, consanguineous, l-serine synthesis, congenital, intrauterine growth restriction, neu laxova syndrome

## Abstract

Neu-Laxova syndrome (NLS) is a rare lethal disorder with autosomal recessive inheritance and is characterized by multiple congenital anomalies. Our case of NLS presented with severe intrauterine growth restriction (IUGR), abnormal facial features, severe central nervous system malformations, skeletal muscle contractures, and the hallmark signs of NLS: ichthyotic skin and excessive subcutaneous tissue with edema. Additionally, testing amniotic fluid from a prior pregnancy with a fetus showing similar abnormalities revealed several regions of homozygosity; one of these regions involved chromosome 1p13.2-p11.2, where the *PHGDH* gene is located. Based on the pattern of findings on serial fetal ultrasounds, postmortem neonatal exams, gross and microscopic exams, radiographs, and genetic analysis in conjunction with the clinical history and the prior pregnancy with the above-described molecular alteration, a final diagnosis of NLS was made. This rare developmental disorder is characterized by heterogenous neuroectodermal defects. Fetal ultrasound in the second trimester can help diagnose it. It is postulated to be caused by loss-of-function mutations in the *PHGDH* (phosphoglycerate dehydrogenase), *PSAT1* (phosphoserine aminotransferase 1), and *PSPH* (phosphoserine phosphatase) genes, which are responsible for de novo L-serine synthesis.

## Introduction

Neu-Laxova Syndrome (NLS) is a rare disorder, with fewer than 100 cases reported in the literature. It is a fatal condition marked by stark abnormalities of tissues derived from the neuroectoderm [1]. The characteristic features include intrauterine growth restriction (IUGR), limb deformities, skin and soft tissue changes, central nervous system malformations, and typical craniofacial features [1]. This disorder results in fetal or neonatal death within hours after delivery [1]. Most reported cases reveal a normal karyotype [1].

## Case presentation

Clinical history

A 26-year-old G8T2P2A4L1 mother with a history of parental consanguinity was receiving prenatal care at our hospital. She had a poor obstetric history, with several pregnancies complicated by fetal anomalies resulting in intrauterine fetal demise (IUFD) and neonatal demise. Fetal ultrasound at 25w3d gestation was concerning due to an overall abnormal profile, including micrognathia, clenched hands and toes, severe IUGR with <3% growth, and the absence of cavum septi pellucidi (a marker for normal central forebrain development, when present). Genetic studies were not completed during this pregnancy. At 35w5d gestation, due to abdominal pain, vaginal bleeding, and decreased fetal movements, she underwent palliative induction and had an uncomplicated normal spontaneous vaginal delivery. A female neonate weighing 860 g was born with IUGR, micrognathia, and microcephaly. The baby’s hospital course was complicated by hypoxemic respiratory failure of unknown etiology and five unsuccessful intubation attempts due to severe micrognathia. Following cardiorespiratory failure with attempted resuscitation, the neonate was placed on ventilation with CPAP (continuous positive airway pressure). Finally, despite maximal intervention, she was moved to the mother’s arms for end-of-life transition and passed away about five hours after birth.

Autopsy evaluation revealed the following

External Exam

**Figure 1:**
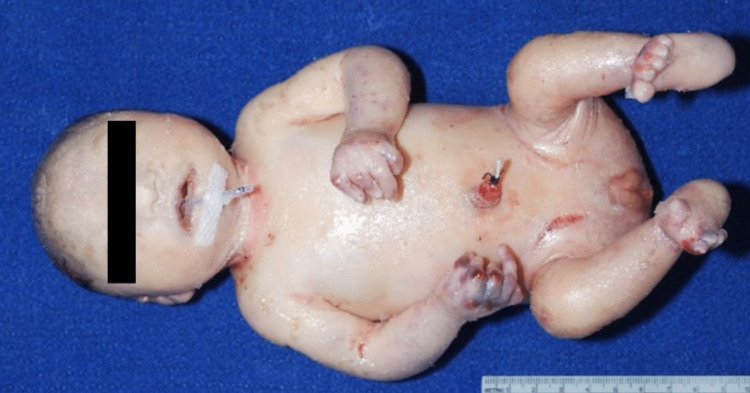
An overall view of the deceased neonate, highlighting severe contractures of the upper and lower extremities, including the digits.

Marked IUGR was seen as evidenced by linear measurements and weights, namely crown-heel length 30.9 cm (mean for age: 44.4 ± 4.4 cm), head circumference 21.3 cm, (mean for age: 31.9 ± 1.6 cm), biparietal diameter 5.7 cm, (mean for age: 8.78 ± 0.52 cm), body weight 833.6 g (mean for age: 2246 ± 511 g), brain weight = 15.9 g (mean for age: 29.2 ± 4.2 g), lungs (combined) weight 10.94 (mean for age: 45.1 ± 12.2 g), liver weight 43.74 g (mean for age: 92.8 ± 22.9 g), kidneys (combined) weight 6.0 g (mean for age: 21.3 ± 6.0 g). The neonate had cutaneous abnormalities, widespread contractures with clenched hands and toes, and flexion of the knees, elbows, and all digits to varying degrees (Figure 1).

**Figure 2:**
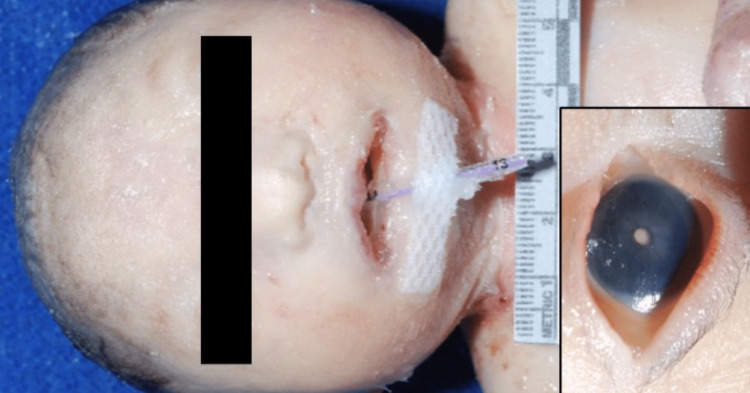
Facial features: microcephaly, hypertelorism, bilateral cataracts (inset), flattened nasal bridge, and severe micrognathia.

**Figure 3:**
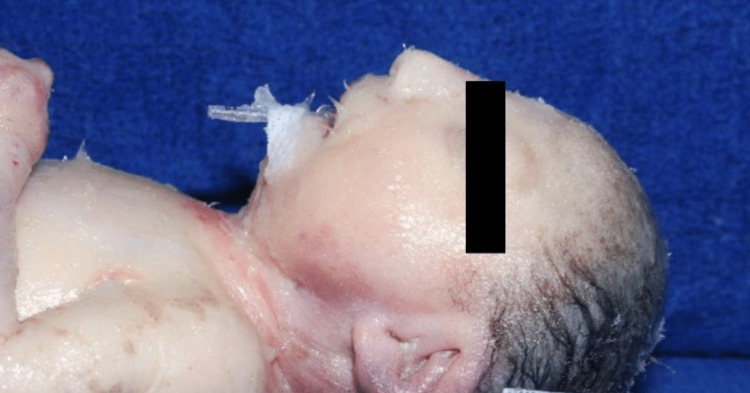
Facial features: microcephaly, flattened nasal bridge, low-set and abnormally formed ears, and severe micrognathia. Petroleum jelly was applied to the skin due to excessive skin cracking.

She also had dysmorphic features, including microcephaly with a sloping forehead, hypertelorism, small palpebral fissures, large low-set abnormally formed ears, a prominent nose with a broad nasal bridge, and micrognathia (Figures 2-3). Also noted were bilateral cataracts and a cleft palate.

**Figure 4:**
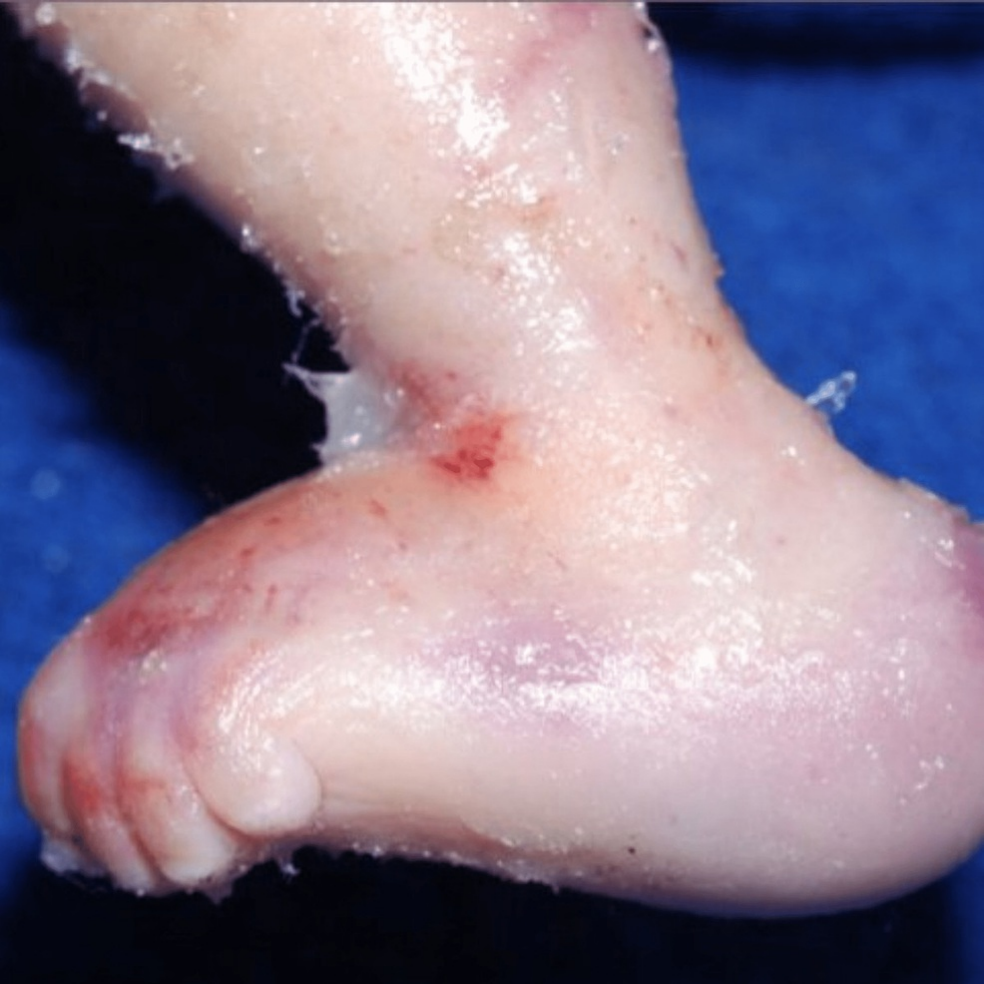
Rocker bottom feet and contractures of the toes.

She had an abnormally formed labia majora and anus positioned close to the genitalia. There were absent palmar creases bilaterally. Other abnormalities of the digits included unusually long fourth fingers bilaterally, sandal gap deformity of the first and second toes bilaterally, syndactyly of the second through fifth toes bilaterally, and bilateral rocker bottom feet (Figure 4). The neonate presented with a hypoplastic gluteal cleft and sacral agenesis.

Internal Exam

The skull was thickened, and the brain was very small for her estimated gestational age, with marked cerebellar hypoplasia and the absence of the corpus callosum.

Microscopic Findings

Histologic examination of the central nervous system showed abnormal/delayed cortical development, abnormal gyration, abnormal cerebellar development, and abnormal white matter/pyramidal tract localization. Sections from the brain showed a three-layer cortex commensurate with the development of a 20-week-old fetus. Her cutaneous abnormalities were confirmed to be ichthyosis on histologic examination, along with excess subcutaneous fat and edema. The kidneys showed intra-tubular calcifications.

Additional Tests

Postmortem radiographs were confirmatory of microcephaly and an S-shaped spine (sacral agenesis). The cause of death was determined to be respiratory failure in the setting of multiple congenital anomalies. 

Ancillary Tests

Karyotypes obtained from fibroblasts cultured from the skin showed no obvious karyotype abnormalities. Fluorescent in situ hybridization (FISH) revealed a female pattern without aneuploidy. Chromosomes 13, 18, 21, X, and Y were probed and found to be normal. The DNA deletion and duplication arrays were negative. The normal findings do not rule out the diagnosis of genetic abnormalities due to mutation types such as point mutations that are not detected with this assay. Further review of maternal prenatal history revealed that loss of heterozygosity (LOH) testing on an amniocentesis specimen from a prior pregnancy revealed a 7.92 Mb region of homozygosity involving chromosome 1p13.2-p11.2, the region of the PHGDH gene.

Based on the pattern of findings on serial fetal ultrasounds, postmortem neonatal exams, gross and microscopic exams, radiographs, and genetic analysis in conjunction with the clinical history and the prior pregnancy with the above-described molecular alteration, a final diagnosis of NLS was made.

## Discussion

Pathophysiology

NLS is a lethal autosomal recessive malformation syndrome. This disorder is characterized by homozygous mutations of the phosphoglycerate dehydrogenase (PHGDH [MIM 606879]) gene, which is involved in the first and limiting step in L-serine biosynthesis, or in other genes encoding enzymes for de-novo L-serine synthesis, namely phosphoserine aminotransferase 1 (PSAT1 [MIM 610936]) and phosphoserine phosphatase (PSPH [MIM 172480]) [2-5]. It was postulated that a lack of adequate serine supply could impair nucleotide synthesis, impair the development of the central nervous system, and affect cell proliferation during embryonic development. These could be the major pathophysiological mechanisms in NLS, culminating in IUGR, microcephaly, and other malformations seen in NLS [5].

Consanguinity

Most described cases have occurred in the setting of consanguineous parentage among diverse ethnicities [6,7], but cases without any history of consanguinity have also been reported [8].

Characteristic Features

Intrauterine growth restriction: This is a common manifestation and is characteristically symmetrical in nature [7,9,10].

Limb Deformities

The limb posture is quite peculiar, with clenched hands, flexed wrists and elbows, hyperextended legs, flexed hips, and the occasional presence of flexion deformities as well as vertebral anomalies like kyphosis [1,11]. It has been postulated that the abnormal development of arteries, nerves, muscles, and bones during embryogenesis is mainly the underlying cause of limb deformities [6,12].

Skin and Soft Tissue

The skin shows a tight appearance with scales and has been described as lamellar ichthyosis [7,13]. The peculiar skin changes could result in limb anomalies, flexion contractures, decreased fetal movements, and IUGR [6,13,14]. The skin changes are also responsible for protein loss through skin fissures and hypoproteinemia, which culminates in massive edema throughout the body [1,15].

Intrauterine Bone CNS and Craniofacial Features

Microcephaly has been reported frequently. Lissencephaly, cerebellar hypoplasia, and an absent or hypoplastic corpus callosum, dilated or abnormal ventricles, and decreased or absent gyri have been observed with decreasing frequency, respectively [6]. CNS abnormalities lead to decreased fetal movements [16]. Characteristic facial appearance is observed due to the presence of a slanting forehead, hypertelorism, proptosis, a flat nose, a nasal bridge, low-set or malformed ears, micrognathia, a cleft lip, and a cleft palate [1,7].

Differential Diagnosis

The differential diagnosis includes a variety of conditions, which have been enumerated in Table 1 [1,6,11].

**Table 1:** Differential diagnosis of Neu Laxova syndrome. CNS: central nervous system, NLS: Neu Laxova syndrome.

Differential diagnosis	Similar features with NLS	Distinguishing features
Cerebro-oculo-facio skeletal syndrome (COFS)	Craniofacial malformations (microcephaly, brain hypoplasia), brain and spinal cord malformations, musculoskeletal defects (flexion contracture, micrognathia)	Typically, normal birth weight, deep-set eyes with blepharophimosis, prominent root of the nose
Pena-Shokeir	Craniofacial malformations, intrauterine growth restriction, pulmonary hypoplasia	Abnormal fetal movement, lack of CNS and skin manifestations
Walker-Warburg Syndrome	Lissencephaly, ventriculomegaly	Can be diagnosed in the first trimester, absence of limb abnormalities, severe subcutaneous edema, and ichthyosis seen in NLS
Multiple pterygium syndrome	Growth retardation, skeletal abnormalities, craniofacial malformations, flexion contractures	Webbing of the skin, pterygia bridging of virtually all joints
Harlequin fetus	Ichthyosis, eclabium, ectropion, limb contractures	Absence of severe microcephaly and CNS abnormalities seen in NLS

Diagnostic Modalities

Prenatal ultrasound monitoring is the only way to diagnose NLS due to the absence of a specific cytogenetic marker [6]. Patients at risk have been recommended for serial ultrasonographic monitoring [9,13]. The peculiar ultrasound findings most frequently seen in NLS include IUGR, flexion deformities, edema, microcephaly, facial features like a sloping forehead, ocular proptosis, hypertelorism, and polyhydramnios. The biparietal diameter has been reported to be consistently decreased in these cases, explaining the underlying microcephaly and other CNS abnormalities. Additional ultrasound findings observed include intracranial abnormalities and the absence of sucking, swallowing, and breathing movements [6,9,16]. Prenatal ultrasound monitoring has been reported to be a reliable method to diagnose NLS and is necessary to consider for making management decisions due to the lethal outcomes of this syndrome despite normal delivery of the fetus in most cases [6].

Prognosis

In the literature, virtually all children with this extremely rare disorder are stillborn or die within hours to weeks after birth; long-term survival beyond six months has not been described.

Management

L-serine supplementation to the mother antenatally and to the child after birth has been reported to accelerate brain growth and psychomotor development in 3-phosphoglycerate-dehydrogenase (3-PGDH) deficiency, an L-serine biosynthesis disorder, thus attenuating the severity of this lethal disease. [17]. Due to the late presentation of the clinical manifestations of this disorder, diagnosis in early pregnancy can be challenging. However, supplement therapy can be attempted in cases with a prior history of NLS [2,5]. Consultation with a clinical geneticist may be of utility in providing further guidance regarding future family planning.

## Conclusions

Neu-Laxova syndrome is a rare, lethal hypokinetic developmental disorder characterized by neuroectodermal defects. Prenatal ultrasound findings like IUGR, flexion deformities, edema, microcephaly, facial features like a sloping forehead, ocular proptosis, hypertelorism, polyhydramnios, and decreased biparietal diameter during the second trimester should prompt clinicians to consider this condition. This syndrome is postulated to be caused by loss-of-function mutations in the *PHGDH*, *PSAT1*, and *PSPH* genes, which are responsible for de novo L-serine synthesis. L-serine supplementation for the mother antenatally and for the child after birth has been reported to attenuate the severity of this condition. There is scope for further research to identify better antenatal diagnostic modalities and treatment options for this condition.
